# Assessing animal welfare impacts of cosmetic manipulations in dromedary camels: insights from oxidative and inflammatory biomarkers

**DOI:** 10.3389/fvets.2026.1720235

**Published:** 2026-03-16

**Authors:** Mohamed Tharwat, Tariq I. Almundarij, Hassan Barakat

**Affiliations:** 1 Department of Clinical Sciences, College of Veterinary Medicine, Qassim University, Buraydah, Saudi Arabia; 2 Department of Medical Biosciences, College of Veterinary Medicine, Qassim University, Buraydah, Saudi Arabia; 3 Department of Food Science and Human Nutrition, College of Agriculture and Food, Qassim University, Buraydah, Saudi Arabia

**Keywords:** acute phase response, animal welfare, biomarkers, cosmetic procedures, dromedary camels, oxidative damage

## Abstract

Cosmetic alterations in camels, such as lip binding, hormone and filler injections, and lip stretching, are common at beauty festivals and severely compromise animal welfare. This study aimed to quantify their physiological effects by analyzing inflammation and oxidative stress biomarkers. A total of 12,385 dromedary camels were examined during the 7th King Abdulaziz Camel Festival (KACF). Camels with identified tampering were classified into five groups: lip binding (LBC), hormone injection (HIC), lip stretching (LSC), perinasal filler injection (PFIC), and lip filler injection (LFIC), with 20 age-matched healthy camels as controls (NC). Serum samples were analyzed for inflammatory markers (serum amyloid A, SAA) and oxidative stress biomarkers [total superoxide dismutase (T-SOD), catalase (CAT), reduced glutathione (GSH), and malondialdehyde (MDA)]. All cosmetic procedures were associated with significant changes in SAA and oxidative-stress biomarkers (T-SOD, CAT, GSH, and MDA), consistent with a systemic inflammatory and oxidative stress response in dromedary camels. SAA levels were highest in HIC (11.82 ng mL^−1^), representing a 33.6% increase versus NC. Antioxidant enzymes showed severe depletion: T-SOD activity decreased by 22.9% in LSC, 27.3% in PFIC, and 25.3% in LFIC groups; CAT activity was most reduced in HIC and PFIC (21.5–23.4% decrease); and GSH levels dropped by 28.1–30.4% in HIC and PFIC groups. Conversely, MDA concentrations increased dramatically, with LSC and LFIC showing elevations of 80.8 to 87.6%. Correlation analysis revealed strong inverse relationships between SAA and antioxidant markers (*r* = −0.757 to −0.790), while principal component analysis clearly separated treatment groups based on their oxidative stress profiles. In conclusion, biomarker analysis reveals that cosmetic alterations in dromedary camels, particularly hormonal and filler injections, induce severe systemic inflammation and oxidative stress. At the same time, mechanical manipulations, such as lip binding and stretching, also elicit significant physiological distress. These findings provide a rapid, robust, and quantitative framework for detecting illegal manipulations in camels, supporting evidence-based regulatory frameworks that protect animal welfare while respecting cultural traditions.

## 1 Introduction

The proliferation of camel beauty festivals has led to widespread “illegal cosmetic manipulations” in dromedary camels, where illegal tampering methods are employed to enhance appearance and increase market value (1–3). These manipulations include mechanical modifications, such as lip stretching (LS) and ear trimming, as well as chemical injections of silicone, fillers, and botulinum toxin, and hormonal treatments with testosterone and growth hormone (1). A comprehensive study of 12,385 Arabian camels revealed that 7.6% showed evidence of tampering, with LS being most common (52.9%), followed by lip binding (LB) (14.7%) and nasal filler injections (12.8%) (4). Detection requires visual assessment and diagnostic imaging (ultrasonography, radiography, and thermography) plus hormonal assays (1, 4–7). These practices raise serious animal welfare concerns, distort market values, undermine cultural integrity, and threaten food security in camel-dependent communities, necessitating coordinated responses involving scientific research, policy enforcement, and community education (1, 5).

Camel beauty contests adhere to codified aesthetic criteria that emphasize natural features, including large heads, long lips that cover the teeth, broad noses, and breed-specific characteristics. However, five primary illegal cosmetic manipulations have emerged: LB using rubber bands to restrict blood flow and cause artificial swelling (identified in 14.3–15% of examined animals); hormone injection (HI) with testosterone and growth hormone causing exaggerated somatic growth, masculinization, and various complications including infertility; LS through daily mechanical manipulation (the most prevalent alteration at 43.4–63.3% of cases); perinasal filler injection (PFI) using silicone and hyaluronic acid-based substances to enhance nose profiles; and lip filler injection (LFI) creating more prominent facial features (found in 4.4–7.9% of examined camels) (1, 3, 4, 8).

Systematic examinations during the 7th King Abdulaziz Camel Festival (KACF) identified 943 animals (7.6%) among 12,385 dromedary camels with confirmed evidence of tampering. At the same time, retrospective analysis from the third through seventh KACF seasons revealed alarming upward trends in all tampering methods, with overall prevalence increasing dramatically from 0.193 to 3.496% (9). Detection requires sophisticated diagnostic approaches, including visual inspection, ultrasonography, radiography, and infrared thermography, with ultrasonography proving particularly valuable for detecting subcutaneous filler materials (4). These practices raise critical animal welfare concerns as they are frequently performed without veterinary oversight, resulting in significant pain, infection risk, impaired biological functions, chronic stress responses, and weakened immune defenses (1, 5).

The economic and societal implications extend beyond immediate animal welfare concerns, creating market distortions by misleading buyers regarding critical animal attributes and artificially inflating prices (1). Legal frameworks, including Saudi Arabia’s Animal Welfare Act and international conventions advocating humane animal treatment, exist but face enforcement gaps and limited veterinary oversight during major events (10). The issue represents a complex intersection of cultural traditions, economic incentives, and ethical responsibilities that demands coordinated scientific, regulatory, and educational approaches to safeguard both animal welfare and market integrity in camel-dependent communities (11, 12).

Illegal cosmetic manipulations lack documented physiological assessment, necessitating biomarker quantification of inflammatory and oxidative stress instead of physical detection alone (1). By measuring established oxidative stress biomarkers such as malondialdehyde (MDA), superoxide dismutase (SOD), catalase (CAT), and reduced glutathione (GSH) (5, 13–16) alongside key inflammatory mediators such as serum amyloid A (SAA) (17), this study will deliver the first biomarker-based assessment of cosmetic-procedure harm. The resulting data will provide objective evidence of animal welfare compromise to inform regulatory enforcement (1), advance welfare science through validated biomarker panels (18), and support the development of quantitative, evidence-based criteria and policies for protecting livestock in cultural and commercial contexts (1, 18). Therefore, the present study aims to comprehensively investigate and determine the effects of various cosmetic practices, including LB, HI, LS, PFI, and LFI, on inflammatory responses and the acceleration of oxidative stress in dromedary camels (*Camelus dromedarius*). The research seeks to establish quantitative biomarker profiles that can objectively assess physiological impact on animal welfare and health status, with direct applications to evidence-based regulatory frameworks. By translating basic physiological science into measurable welfare indicators, this work provides policymakers, veterinary professionals, and community stakeholders with objective information to develop protective guidelines that balance animal welfare with respect for cultural traditions, establishing a model applicable to other culturally significant livestock practices worldwide.

## 2 Materials and methods

### 2.1 Study design and sampling

Study design has been reported recently (4). Briefly, A total of 12,385 dromedary camels (*C. dromedarius*) – 12,080 females and 305 males – were examined for signs of tampering during the 7th KACF, approximately 90 km from Riyadh, Saudi Arabia, between December 28, 2022, and January 12, 2023. All camels were healthy and ranged in age from 1 to 20 years. Camel owners voluntarily enrolled their animals in the festival competition, which included mandatory veterinary health assessment and examination for evidence of tampering as a condition of participation. The camels first underwent virtual screening, followed by further examination of suspected tampering cases using ultrasound, thermography, radiography, and biochemical analysis of testosterone and growth hormone levels. Camels identified with tampering were classified into five groups: (1) lip binding camels (LBC), (2) hormone injection camels (HIC), (3) lip stretching camels (LSC), (4) perinasal filler injection camels (PFIC), and (5) lip filler injection camels (LFIC). Twenty age-matched, clinically healthy camels were used as a control group, normal camels (NC). From both tampered and healthy camels, 7 mL blood samples were collected via jugular venipuncture, allowed to clot at room temperature for 30 min, and then centrifuged at 1,500 × *g* for 10 min at 4 °C. The resulting aliquots were stored at −80 ± 1 °C for future analysis.

### 2.2 Serum analyses

#### 2.2.1 Serum amyloid A (SAA)

A commercial camel-specific SAA ELISA kit (Sunlongbiotech, SN: SL0059Cm) was used according to the manufacturer’s instructions. All reagents and samples were equilibrated to room temperature, and a standard curve was generated by serial dilution of recombinant SAA (0.15–2.7 ng mL^−1^). In duplicate wells, 100 μL of standards or diluted serum samples (1:5 to 1:10 dilution) were incubated at 37 °C for 60 min and then washed five times. Samples were incubated with 100 μL HRP-conjugated anti-SAA antibody at 37 °C for 30 min, rewashed, and incubated with TMB substrate (10 min in the dark). The reaction was stopped with 100 μL of 0.2 M H₂SO₄, and absorbance was measured at 450 nm. Sample concentrations were interpolated from the standard curve using a four-parameter logistic fit and adjusted for dilution, with intra-assay CVs maintained below 10% and recoveries ranging from 85 to 115%. Continuous quality controls, provided by the kit and blank wells, were included to ensure assay validity. All procedures adhered to institutional biosafety and waste disposal guidelines.

#### 2.2.2 Total superoxide dismutase (T-SOD)

The T-SOD activity in camel serum was measured using a colorimetric assay kit (Elabscience Biotechnology Inc., Houston, Texas, United States). Briefly, serum samples were thawed on ice, diluted 1:10 in dilution buffer, and pre-incubated with reaction buffer containing xanthine oxidase substrate at 25 °C for 10 min. Standards and diluted samples (20 μL) were added in duplicate to a 96-well plate, followed by 200 μL of working solution and 20 μL of enzyme working solution. The plate was then incubated at 37 °C for 20 min. The reaction was halted by adding 50 μL of stop solution, and absorbance was measured at 450 nm. A standard curve was generated using known SOD units provided in the kit (0–100 U mL^−1^), and sample activities were calculated by interpolation and expressed as U mL^−1^ of serum, with intra- and inter-assay coefficients of variation maintained below 10% and recoveries between 90 and 110% to ensure assay precision. Continuous quality controls and blank wells were included according to the manufacturer’s protocol, and all procedures adhered to institutional safety and waste management guidelines.

#### 2.2.3 Catalase (CAT)

Catalase activity in camel serum was determined using a spectrophotometric method based on hydrogen peroxide decomposition kit (Elabscience Biotechnology Inc., Houston, Texas, United States), with serum samples thawed on ice and diluted 1:20 in phosphate buffer (50 mM, pH 7.0); 50 μL of each diluted sample or standard (0–100 U L^−1^ bovine catalase) was mixed with 950 μL of freshly prepared 10 mM H₂O₂ in a quartz cuvette. The decrease in absorbance at 240 nm was recorded spectrophotometrically at 25 °C for 60 s, with initial linear rates used for activity calculation. Catalase activity was expressed as U L^−1^ of serum, calculated using the molar extinction coefficient of H₂O₂ (43.6 M^−1^ cm^−1^) and taking into account sample dilution. All measurements were performed in triplicate with intra- and inter-assay coefficients of variation maintained below 8% to ensure precision.

#### 2.2.4 Reduced glutathione (GSH)

The GSH levels in camel serum were determined using a colorimetric assay (Elabscience Biotechnology Inc., Houston, Texas, United States) based on the formation of a yellow-colored product upon reaction with 5,5′-dithiobis (2-nitrobenzoic acid) (DTNB). Serum samples were thawed, diluted appropriately, and mixed with DTNB reagent. The mixture was then incubated at room temperature, allowing GSH to reduce DTNB to 5-thio-2-nitrobenzoic acid, which was quantified by measuring the absorbance at 420 nm using a spectrophotometer. GSH concentrations were calculated from a calibration curve prepared with known GSH standards and expressed as mg GSH L^−1^. Assay precision was ensured by running all samples and standards in duplicate, maintaining intra- and inter-assay variations below 10%. Quality control samples with known GSH concentrations were included to validate assay performance, and all procedures were conducted in accordance with institutional biosafety and waste disposal protocols (19).

#### 2.2.5 Malondialdehyde (MDA)

The MDA levels in camel serum were quantified using the thiobarbituric acid reactive substances (TBARS) assay (Elabscience Biotechnology Inc., Houston, Texas, United States); briefly, serum samples were thawed on ice and mixed 1:1 (v/v) with 0.67% thiobarbituric acid in 20% trichloroacetic acid, heated in a boiling water bath for 15 min, cooled on ice, and centrifuged at 3,000 *×* g for 10 min; 200 μL of the supernatant was transferred to a 96-well plate and absorbance was measured at 532 nm. MDA concentrations were calculated by interpolation against a malondialdehyde bis-(dimethyl acetal) standard curve (0–10 nM) prepared in the same reagent matrix, and results were expressed as nmol MDA per mL of serum. All samples and standards were run in duplicate, with intra- and inter-assay coefficients of variation maintained below 10%. Blank wells containing reagents only were included to correct for background absorbance. Continuous quality controls with known MDA concentrations ensured assay validity, and procedures adhered to institutional biosafety and waste disposal guidelines.

### 2.3 Statistical analysis

Statistical analysis was performed using SPSS (V. 22.0, IBM, Houston, Texas). Prior to parametric testing, the normality of data distribution was assessed using the Shapiro–Wilk test for each treatment group; a *p*-value greater than 0.05 was interpreted as indicating that the data met the normality assumptions. Homogeneity of variance was evaluated using Levene’s test. Data that satisfied normality assumptions (*p* > 0.05 in the Shapiro–Wilk test) were analyzed using one-way analysis of variance (ANOVA) with *post hoc* Tukey’s honestly significant difference (HSD) test as described by Steel (20). For data violating normality assumptions, the non-parametric Kruskal-Wallis H test was applied. Results are reported with the F-statistic and degrees of freedom for parametric tests. Statistical significance was established at *α* = 0.05 (two-tailed). Data are presented as mean ± standard error (SE). Pearson’s correlation analysis was used to assess linear relationships between variables, with correlation coefficients and exact *p* values reported. Principal component analysis (PCA) was performed to identify underlying patterns and relationships within the dataset.

## 3 Results

### 3.1 The SAA level

**Figure 1:**
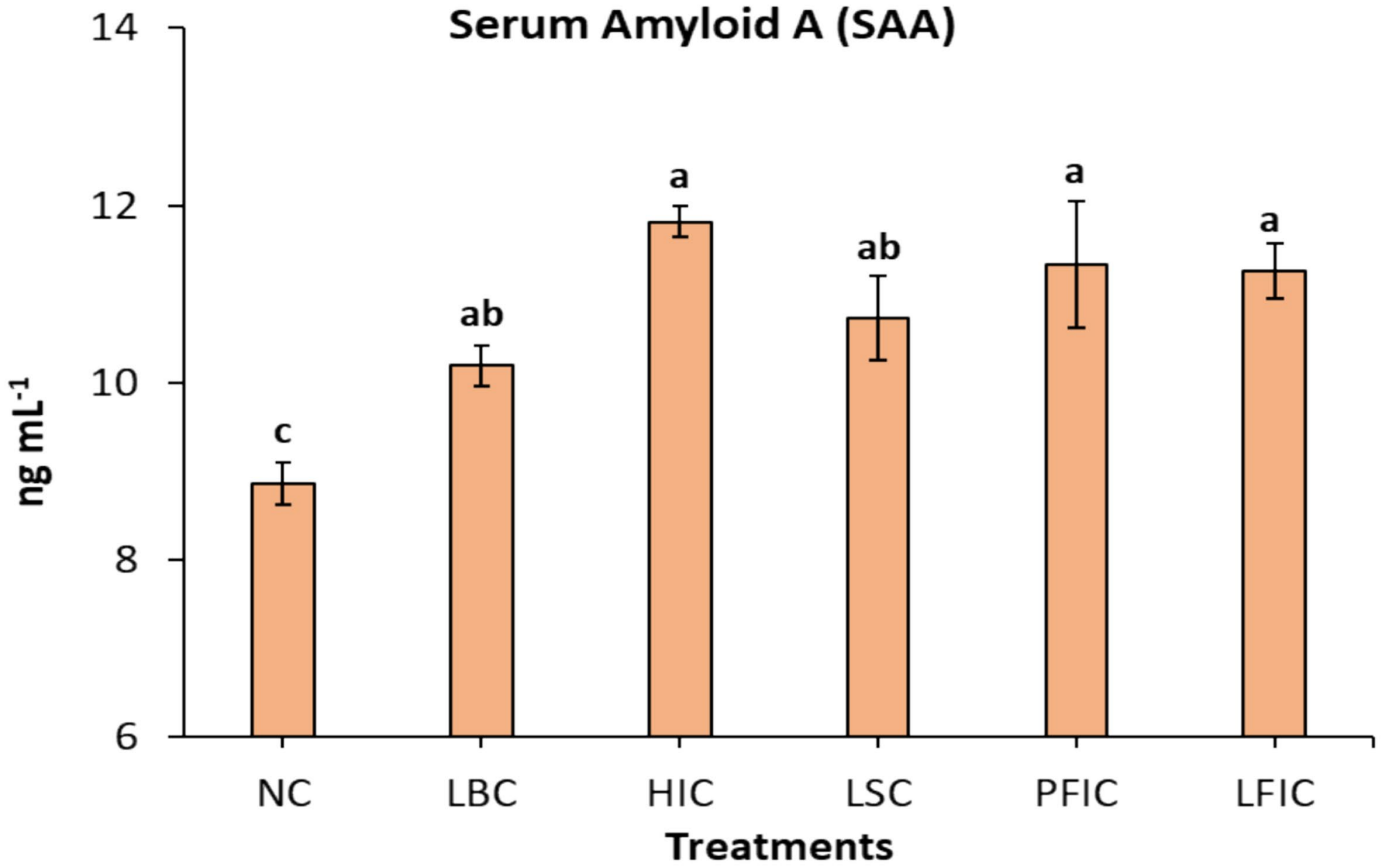
Serum amyloid A (SAA) concentrations in camels subjected to different cosmetic procedures (mean ± SE). Serum SAA levels (ng mL^−1^) were measured in camels following various treatments: NC, normal camels; LBC, lip binding camels; HIC, hormone injection camels; LSC, lip stretching camels; PFIC, perinasal filler injection camels; and LFIC lip filler injection camels). Data are presented as mean ± standard error. ^a,b,c^Bars topped with different letters indicate statistically significant differences between treatments (*p*-value, 0.003).

The serum amyloid A (SAA) levels varied significantly among different treatment groups of dromedary camels subjected to various illegal cosmetic manipulation procedures (Figure 1). Control camels (NC) showed the lowest SAA at 8.85 ng mL^−1^. Hormone-injected camels (HIC) showed the highest at 11.82 ± 0.4 ng mL^−1^ (33.6% increase), followed by perinasal filler (PFIC) and lip filler (LFIC) groups. Lip-binding (LBC) and lip-stretching (LSC) camels showed moderate elevations (10.19 and 10.73 ng mL^−1^; 15.1 and 21.2% increases, respectively). Statistical analysis revealed significant differences among the treatment groups, with the HIC group showing the most pronounced elevation, followed by the PFIC and LFIC groups. At the same time, LBC demonstrated the smallest but still significant increase in SAA concentrations.

### 3.2 The T-SOD level

**Figure 2:**
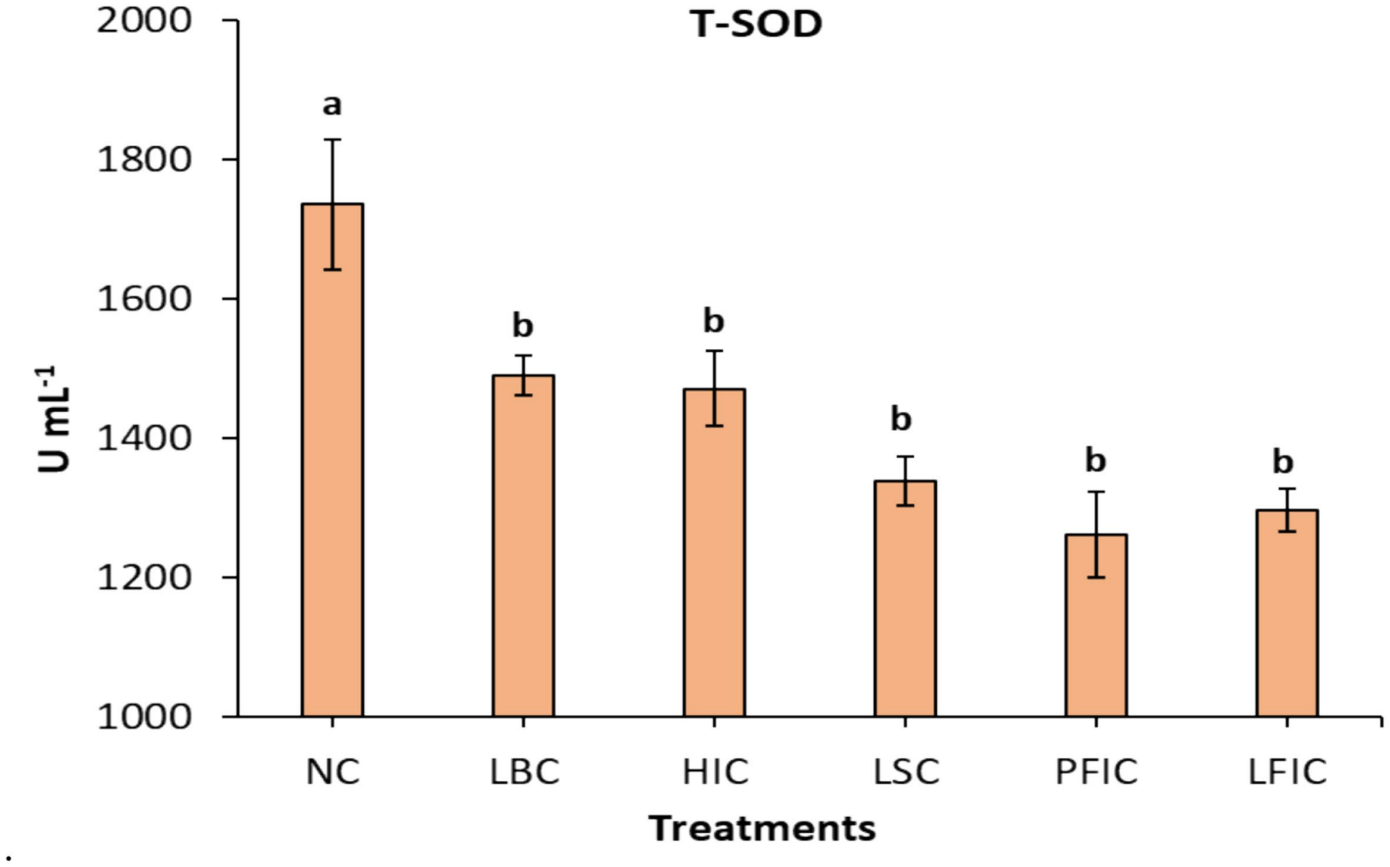
Serum T-SOD activity in camels subjected to different cosmetic procedures (mean ± SE). Serum T-SOD activity (U mL^−1^) was measured in camels following various treatments: NC, normal camels; LBC, lip binding camels; HIC, hormone injection camels; LSC, lip stretching camels; PFIC, perinasal filler injection camels; and LFIC, lip filler injection camels. Data are presented as mean ± standard error. ^a,b^Bars topped with different letters indicate statistically significant differences between treatments (*p* value < 0.0001).

Control camels (NC) exhibited the highest T-SOD activity at 1735 U mL^−1^, significantly elevated compared to all treatment groups (*p* < 0.05). Among treated groups, lip-stretching (LSC), perinasal filler-injection (PFIC), and lip filler-injection (LFIC) camels demonstrated the lowest T-SOD activity, with values of 1,337, 1,262, and 1,297 U mL^−1^, respectively. These represented reductions of 22.9, 27.3, and 25.3% relative to controls. The LBC and HIC groups had intermediate decreases of 14.1 and 15.3%, respectively. The statistical analysis classified the groups into 3 groups: (NC), (LBC and HIC), and (LSC, PFIC, and LFIC) (Figure 2).

### 3.3 The CAT level

**Figure 3:**
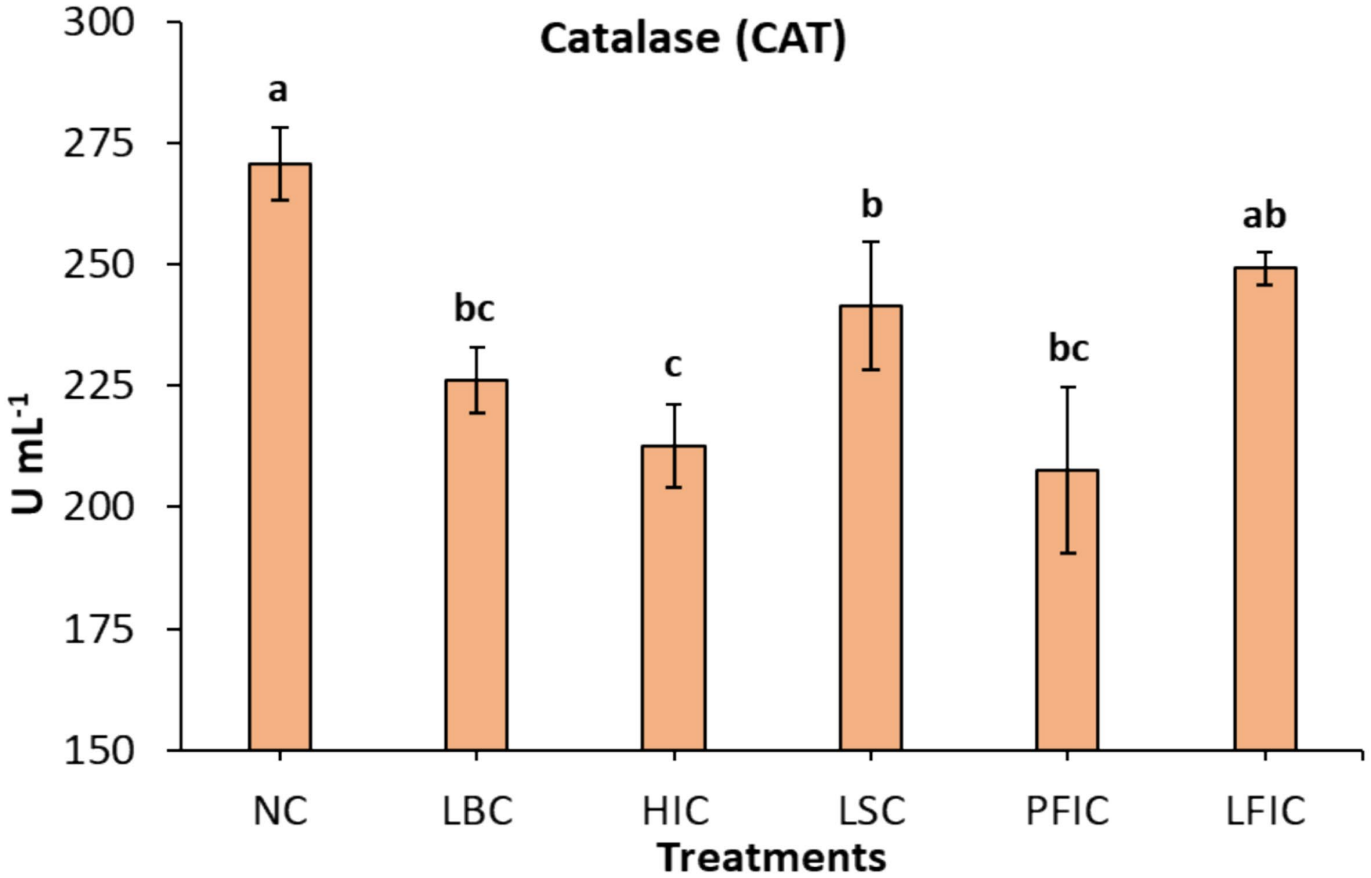
Serum CAT activity in camels subjected to different cosmetic procedures (mean ± SE). Serum CAT activity (U mL^−1^) was measured in camels following various treatments: NC, normal camels; LBC, lip binding camels; HIC, hormone injection camels; LSC, lip stretching camels; PFIC, perinasal filler injection camels; and LFIC, lip filler injection camels. Data are presented as mean ± standard error. ^a,b,c^Bars topped with different letters indicate statistically significant differences between treatments (*p*-value < 0.0001).

The CAT activity in camel serum differed significantly among treatment groups (Figure 3). The NC group exhibited the highest CAT level (270.88 U mL^−1^), which was significantly greater than in all treated groups (*p* < 0.05). The LSC and LFIC groups exhibited a moderate reduction in CAT activity when presented with concentrations of 241.54 and 249.22 U mL^−1^. In contrast, the HIC and PFIC groups exhibited the lowest activities at 212.73 and 207.59 U mL^−1^, respectively. The LBC procedures had intermediate CAT levels of 226.22 U mL^−1^. The statistical analysis applying *post hoc* comparisons classified the groups into 3 groups: NC, (LSC, LFIC, and LBC), and (HIC and PFIC).

### 3.4 The GSH level

**Figure 4:**
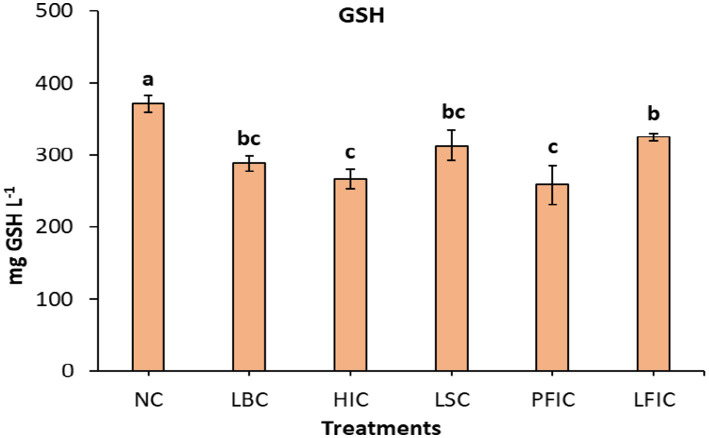
Serum GSH level in camels subjected to different cosmetic procedures (mean ± SE). Serum CAT activity (mg GSH mL^−1^) was measured in camels following various treatments: NC, normal camels; LBC, lip binding camels; HIC, hormone injection camels; LSC, lip stretching camels; PFIC, perinasal filler injection camels; and LFIC, lip filler injection camels. Data are presented as mean ± standard error. ^a,b,c^Bars topped with different letters indicate statistically significant differences between treatments (*p*-value < 0.0001).

The GSH concentrations in camel serum differed significantly among treatment groups (Figure 4). The NC group exhibited the highest GSH level (370.92 mg L^−1^), which was significantly elevated versus all treatment groups (*p* < 0.05). The LFIC and LSC showed the next highest GSH concentration, 324.91 and 312.80 mg L^−1^, respectively. Moderate reductions were observed in the LBC group, exhibiting 288.46 mg L^-1,^ while ^the^ PFIC and HIC groups showed the lowest GSH level, corresponding to a 30.40 and 28.12% decrease relative to NC.

### 3.5 The MDA level

**Figure 5:**
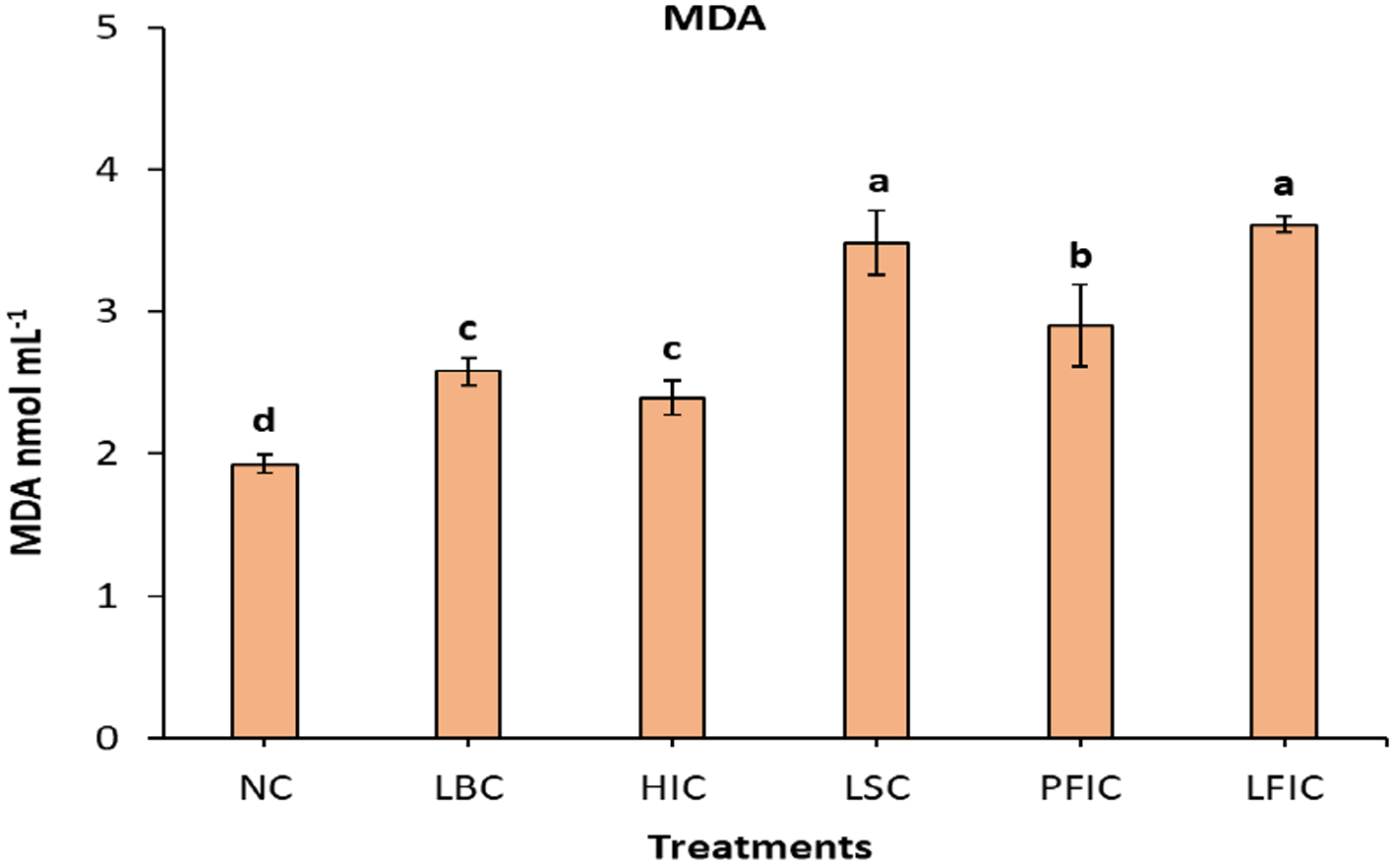
Serum MDA level in camels subjected to different cosmetic procedures (mean ± SE). Serum MDA (nmol mL^−1^) was measured in camels following various treatments: NC, normal camels; LBC, lip binding camels; HIC, hormone injection camels; LSC, lip stretching camels; PFIC, perinasal filler injection camels; and LFIC, lip filler injection camels. Data are presented as mean ± standard error. ^a,b,c^Bars topped with different letters indicate statistically significant differences between treatments (*p*-value < 0.0001).

Malondialdehyde (MDA) concentrations in camel serum differed significantly among treatment groups (Figure 5). The NC group exhibited the lowest MDA level (1.93 nmol mL^−1^), which was significantly lower than all treated groups (*p* < 0.05). The LSC and LFIC groups showed the highest MDA concentration (3.49 and 3.62 nmol mL^−1^), representing an (80.83 and 87.56%) increase versus NC. The PFIC group had moderately elevated MDA levels (2.90 nmol mL^−1^), while the LBC and HIC groups demonstrated lower rises at 2.39 and 2.58 nmol mL-1, corresponding to 23.83 and 33.68% increases, respectively.

Pearson correlation analysis revealed several significant relationships among SAA, T-SOD, CAT, GSH, and MDA in different treated camels. SAA displayed strong negative correlations with CAT (*r* = −0.757), T-SOD (*r* = −0.790), and GSH (*r* = −0.788), and a moderate positive correlation with MDA (*r* = 0.520), all of which were statistically significant (*p* < 0.05). CAT was strongly and positively correlated with GSH (*r* = 0.997) and moderately with T-SOD (*r* = 0.556), while T-SOD also positively correlated with GSH (*r* = 0.604) and showed a strong negative correlation with MDA (*r* = −0.851). MDA showed weak and non-significant correlations with CAT (*r* = −0.086) and GSH (*r* = −0.153). These results indicate robust interconnectedness between inflammation (SAA) and oxidative stress markers (CAT, T-SOD, GSH, MDA), with most variables showing strong relationships except the links with MDA, which were less consistent (Table 1).

**Table 1:** Pearson’s correlation coefficient among the presented analyses for inflammation and oxidative stress biomarkers.

Variables	SAA	CAT	T-SOD	GSH	MDA
SAA	1	−0.757**	−0.790**	−0.788**	0.520*
CAT	−0.757**	1	0.556*	0.997**	−0.086
T-SOD	−0.790**	0.556*	1	0.604*	−0.851**
GSH	−0.788**	0.997**	0.604*	1	−0.153
MDA	0.520*	−0.086	−0.851**	−0.153	1

SAA, serum amyloid A; CAT, catalase enzyme; T-SOD, total superoxide dismutase; GSH, reduced glutathione; MDA, malondialdehyde. Asterisks indicate statistical significance of the Pearson correlation coefficients (two-tailed): *p < 0.05, **p < 0.01.

**Figure 6:**
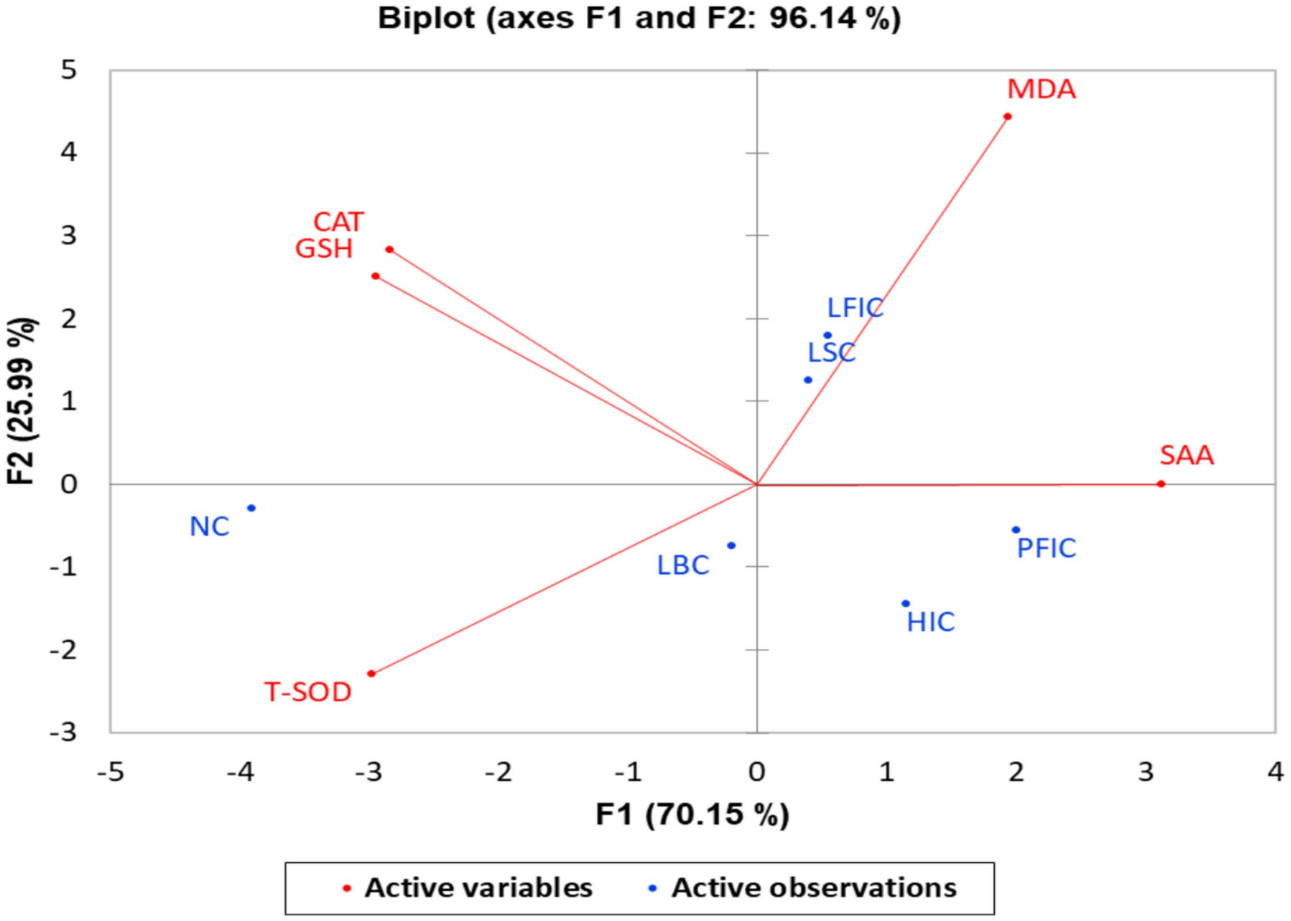
Principal component analysis biplot of oxidative-stress biomarkers in camel treatment groups. GSH (reduced glutathione), CAT (catalase), T-SOD (total superoxide dismutase), MDA (malondialdehyde), and an inflammatory biomarker, SAA (serum amyloid A). The biplot displays active variables (red vectors: MDA, SAA, CAT, GSH, T-SOD) and active observations (blue points) representing camel groups: NC, normal camels; LBC, lip binding camels; HIC, hormone injection camels; LSC, lip stretching camels; PFIC, perinasal filler-injection camels; and LFIC, lip filler injection camels. The first two principal components (F1 and F2) explain 70.15 and 25.99% of the total variance, respectively. Vectors indicate the direction and strength of each biomarker’s contribution to the component axes. The spatial positioning of treatment groups reflects a welfare severity gradient: LFIC (lip filler injection camels) and LSC (lip stretching camels) occupy the PCA space farthest from NC (normal camels), indicating the most severe combined systemic inflammation and oxidative damage. PFIC (perinasal filler injection) and HIC (hormone injection camels) show intermediate separation, reflecting substantial but less severe physiological burden. LBC (lip binding camels) occupies a transitional position. NC is strongly isolated in the high antioxidant-enzyme quadrant, representing baseline healthy status. This visualization enables the rapid assessment of treatment severity and welfare impact based on the distance from the control group.

**Figure 7:**
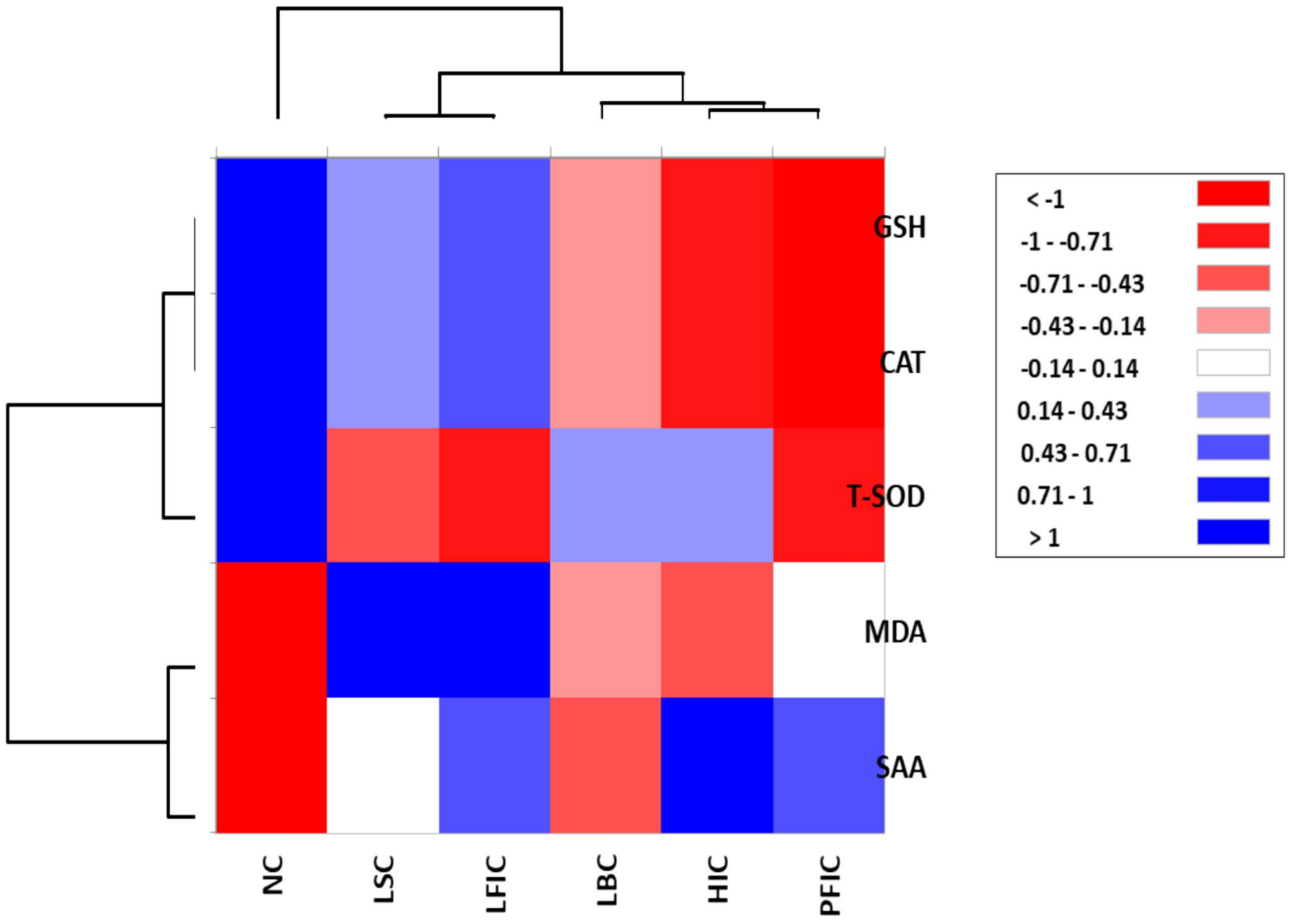
Heatmap and hierarchical clustering of oxidative stress biomarkers across camel treatment groups. Rows represent biomarkers: GSH (reduced glutathione), CAT (catalase), T-SOD (total superoxide dismutase), MDA (malondialdehyde), and an inflammatory biomarker, SAA (serum amyloid A). Columns represent camel groups: NC, normal camels; LBC, lip binding camels; HIC, hormone injection camels; LSC, lip stretching camels; PFIC, perinasal filler injection camels; and LFIC, lip filler injection camels. Color intensity reflects standardized values (*z*-scores), with deep red indicating low values (< −1), white near zero (−0.14 to 0.14), and deep blue indicating high values (>1). Dendrograms depict hierarchical clustering of both biomarkers and treatment groups based on Euclidean distance and complete linkage. Color gradients quantify animal welfare compromise. Blue coloration (elevated SAA, MDA; low antioxidants) indicates severe welfare compromise, while red (indicating antioxidant depletion) indicates stress-induced depletion. White indicates a near-normal baseline. Column clustering reveals a welfare severity hierarchy: NC (left) exhibits optimal protective capacity; LSC and LFIC (center-right) demonstrate severe combined inflammatory-oxidative stress; PFIC and HIC (right) show the most severe physiological compromise. Distance-based welfare ranking enables an objective assessment of the impact of procedures and supports regulatory decision-making.

Multivariate analyses directly reflect physiological welfare compromise, with spatial distance from control animals serving as a quantitative indicator of welfare severity. Groups positioned furthest from NC control exhibit the most severe systemic inflammation-oxidative stress and the most significant welfare impairment. Group separation in multivariate visualizations (Figures 6, 7) directly reflects differential physiological welfare compromise. Animals positioned furthest from the NC (control) group in PCA space exhibit the most severe combined inflammatory and oxidative stress burden, indicating maximum welfare impairment. Heatmap color gradients (blue = elevated stress markers/depleted antioxidants) quantify deviations from a healthy baseline, enabling the objective stratification of welfare severity. This approach translates complex biomarker relationships into interpretable welfare categories supporting evidence-based assessment and regulatory decision-making.

Interestingly, principal component analysis (PCA) of all measured variables clearly separated the different treatment groups of camels based on their inflammatory and oxidative stress profiles (Figure 6). The first principal component (F1, 70.15%) primarily distinguished groups with high SAA and MDA levels from those with elevated antioxidant enzyme levels (CAT, GSH, T-SOD). LFIC and LSC grouped closely with maximal MDA and SAA scores, representing the most severe combined inflammatory and oxidative response among all treatments. PFIC and HIC displayed intermediate separation, which was associated with high SAA and moderate MDA values but with relatively lower antioxidant activities. NC was strongly separated from all other groups, characterized by high CAT, GSH, and T-SOD and minimal inflammation or oxidative stress. At the same time, LBC occupied a transitional position between control and intensively treated animals. The second principal component (F2, 25.99%) contributed further to the differentiation, especially between groups with highly depleted antioxidants (CAT, GSH) and high oxidant/acute phase markers (MDA, SAA).

The hierarchical clustering heat map revealed distinct patterns of biomarker expression across treatment groups and variables (Figure 7). The dendrogram clearly separates antioxidant markers (GSH, CAT, T-SOD) from stress indicators (MDA, SAA), with treatment groups clustering according to their physiological profiles. The NC displayed the highest normalized values for all antioxidant enzymes (deep red coloration for GSH, CAT, and T-SOD) and the lowest values for stress markers (deep blue for MDA and SAA). LSC and LFIC groups showed intermediate antioxidant depletion with moderate negative values (light blue to white) for protective enzymes, while exhibiting elevated stress markers. HIC and PFIC demonstrated severe antioxidant compromise (deep blue for GSH, CAT, T-SOD) coupled with maximal inflammatory and oxidative stress responses (deep red for SAA and MDA). LBC occupied an intermediate position, characterized by moderate depletion of antioxidants and mild elevation of stress markers. The clustering analysis grouped GSH and CAT together as the most closely related antioxidant markers, while SAA formed a distinct cluster reflecting its role as an acute-phase inflammatory indicator.

## 4 Discussion

While this investigation provides the first comprehensive biomarker-based assessment of the effects of cosmetic tampering on dromedary camel welfare, several contextual factors merit consideration and offer clear opportunities for future research advancement. However, reliable detection of illegal cosmetic manipulation requires integrating multiple approaches (10, 11, 18). Biomarkers (SAA, antioxidant enzymes) reveal subclinical inflammation and oxidative stress, while noninvasive imaging (ultrasonography, infrared thermography) localizes fillers without additional trauma (3). Molecular assays confirm the presence of hormone residues or foreign substances. This integrated protocol enhances diagnostic accuracy and reproducibility in veterinary settings (21). Elevated SAA levels across all treatment groups confirm systemic inflammation from cosmetic manipulations. SAA, a major acute-phase protein, increases dramatically within 24–48 h following tissue injury. The differential SAA responses among treatment groups reflect the varying degrees of tissue trauma and inflammatory burden imposed by different cosmetic procedures (22, 23). However, the highest SAA elevation in the HIC group suggests that hormonal manipulation, particularly involving testosterone and growth hormone administration, creates the most severe systemic inflammatory response. This finding aligns with previous research demonstrating that hormonal stress significantly influences the production of acute-phase proteins and activation of the immune system. SAA elevation reflects the combined effects of tissue trauma and endocrine-induced metabolic stress. Altered endocrine function exacerbates oxidative damage and inflammatory cascades (17, 24). Similarly, significant SAA elevations in filler injection groups (PFIC and LFIC) reflect the inflammatory response to the introduction of foreign material, consistent with reports of acute-phase responses following cosmetic procedures involving subcutaneous injections. Silicone and hyaluronic acid fillers trigger local inflammatory reactions. These manifestations are systemic, resulting from increased hepatic SAA synthesis, which is mediated by cytokines IL-6 and TNF-α. This inflammatory cascade represents the body’s attempt to respond to perceived tissue damage and the presence of foreign substances (25–29). In the same context, SAA elevation in the LBC and LSC groups demonstrates that mechanical manipulation alone is sufficient to trigger systemic inflammatory responses, causing direct tissue trauma through restricted blood flow, mechanical stress, and associated pain responses, which lead to cytokine release and subsequent acute-phase protein synthesis (30, 31). Indeed, the consistent pattern of SAA elevation across all treatments provides objective biochemical evidence of the physiological burden imposed by illegal cosmetic manipulations, supporting animal welfare concerns regarding these practices. The SAA response serves as a sensitive biomarker for detecting and quantifying the inflammatory impact of aesthetic manipulations, offering a more objective assessment tool than purely visual inspection methods. These findings align with established principles of the surgical stress response, where the magnitude of tissue trauma directly correlates with elevation of acute-phase proteins (23, 32–34). The dynamics of SAA reveal a multifaceted interaction among tissue damage, cytokine-mediated signaling, and hepatic acute-phase activation, collectively typifying the organism’s response to physiological insults and stress. Within camel welfare evaluation frameworks, SAA quantification provides critical insight into the biological consequences of cosmetic procedures, thereby reinforcing regulatory initiatives aimed at eliminating these detrimental practices while establishing an empirical basis for science-driven welfare protocols (22, 23).

T-SOD activity declined significantly, indicating compromised antioxidant defense. SOD catalyzes the superoxide dismutation reaction to produce hydrogen peroxide. Its depletion signifies increased ROS burden and impaired redox homeostasis (35). The reduction in the LBC and HIC group suggests that hormonal manipulation exacerbates oxidative damage, likely due to endocrine-induced metabolic stress combined with injection trauma (36). The most significant T-SOD decreases in PFIC groups are likely due to mechanical tissue damage and the introduction of foreign substances. This aligns with reports that repetitive stress and filler injections reduce the activities of antioxidant enzymes (37). The comparatively lower T-SOD activities in the LFIC and LSC groups suggest that oxidative challenges are higher in lip-specific and combined mechanical procedures compared to hormonal and lip-binding interventions. These findings underscore the utility of T-SOD as a sensitive biomarker of oxidative stress in camels subjected to cosmetic tampering, highlighting the significant physiological burden of these practices and reinforcing the need for objective welfare assessments that incorporate antioxidant status (1).

The observed decrease in CAT activity across all illegal cosmetic manipulation treatment groups indicates a compromised antioxidant defense in response to tissue trauma and systemic stress induced by these procedures. Catalase, a key enzyme in hydrogen peroxide detoxification, is known to decline under oxidative challenge, reflecting increased reactive oxygen species (ROS) burden and impaired redox homeostasis (38). The most significant decline in catalase activity within the HIC and PFIC cohorts indicates that hormonal manipulation and filler injection procedures induce more severe oxidative stress, presumably attributable to synergistic metabolic disruptions and inflammatory responses triggered by exogenous materials. The modest reductions observed in the LBC and LSC groups additionally confirm that mechanical interventions independently provoke substantial oxidative stress, aligning with documented evidence that repeated tissue distension and ischemia–reperfusion cycles diminish antioxidant enzyme functionality (39). The relatively higher CAT activity in LFIC compared to HIC and PFIC may reflect differential tissue responses to lip-specific filler injections versus perinasal or hormonal interventions (40, 41). Overall, these findings corroborate the role of CAT as a sensitive biomarker of oxidative damage and highlight the substantial physiological burden of illegal cosmetic manipulations in camels, underscoring the need for objective welfare assessments integrating antioxidant status measurements (42–44).

The marked decline in GSH levels across all groups of camels undergoing illegal cosmetic manipulation indicates depletion of a critical intracellular antioxidant, reflecting significant oxidative stress induced by these aesthetic manipulations. GSH serves as a primary non-enzymatic defense against reactive oxygen species (ROS), and its reduction signifies an increased oxidative burden and impaired detoxification capacity (39). The most pronounced GSH depletion in the PFIC group suggests that PFI provokes the greatest oxidative challenge, likely due to intense localized inflammation and prolonged foreign body reactions (45). The HIC group’s substantial GSH decrease further underscores the compounded oxidative effects of hormonal manipulation and injection trauma, aligning with reports that exogenous hormone administration disrupts redox balance and reduces antioxidant reserves (46). The comparatively elevated glutathione concentrations in LFIC and LSC cohorts suggest that although mechanical procedures also impose oxidative challenges, their deleterious effects remain less pronounced than those induced by chemical or hormonal manipulations. These results substantiate GSH quantification as a sensitive indicator for identifying oxidative perturbations in camels exposed to aesthetic modifications, thereby strengthening the imperative for thorough assessment of antioxidant capacity within animal welfare evaluation protocols (47).

The significant elevation of MDA across all groups of camels subjected to illegal cosmetic manipulation indicates enhanced lipid peroxidation and oxidative damage. MDA, a reactive aldehyde product of polyunsaturated fatty acid peroxidation, serves as a reliable marker of cellular membrane injury under oxidative stress (48). The highest MDA levels in LSC and LFIC groups suggest that combined mechanical stretching with filler injections causes the most severe lipid peroxidation, likely due to repetitive tissue trauma and foreign material–induced inflammation (40). The elevated malondialdehyde concentrations observed in the PFIC and LBC cohorts further corroborate that both chemical and mechanical interventions perturb the redox equilibrium, aligning with evidence from other species that demonstrates ischemia–reperfusion events and injection-mediated procedures elevate lipid peroxidation indices. The relatively diminished MDA levels in HIC compared with LSC and LFIC may indicate that hormonal administration imposes predominantly metabolic, rather than exclusively mechanical, stress, triggering oxidative cascades via endocrine system disruption (49). Overall, these results validate MDA measurement as a sensitive indicator of oxidative injury in camels subjected to cosmetic tampering and emphasize the substantial physiological burden of these practices on animal health and welfare.

Correlation analysis showed strong inverse relationships between SAA and antioxidant markers (CAT, T-SOD, and GSH). This confirms that an increasing inflammatory burden is associated with reduced antioxidant capacity (48). The positive association between SAA and MDA suggests that systemic inflammation parallels increased lipid peroxidation and cellular damage, in line with literature on acute-phase response and oxidative injury during animal stress and trauma (50). The near-perfect positive correlation between CAT and GSH underscores the tight co-regulation and compensatory response of the enzymatic and non-enzymatic antioxidant systems under stress, agreeing with previous studies in camelids and other livestock on coordinated antioxidant defense (13). The moderately strong positive associations between T-SOD and both CAT and GSH further reinforce the existence of a coordinated antioxidant defense system, demonstrating that SOD activity exhibits parallel dynamics with other redox indicators to counteract reactive oxygen species-induced injury. The robust inverse relationships between T-SOD and MDA, coupled with the absence of significant correlation between MDA and either CAT or GSH, suggest that superoxide-mediated lipid peroxidation represents a particularly pivotal consequence of cosmetic manipulations, while other antioxidant systems become differentially exhausted or overburdened under severe oxidative stress. Collectively, these findings underscore that inflammatory activation and oxidative stress are intimately interconnected in camels subjected to aesthetic modifications, thereby endorsing biomarker-driven methodologies for welfare assessment (13).

The PCA revealed distinct clustering of treatment groups based on inflammatory and oxidative stress profiles. LFIC and LSC occupied PCA space furthest from control, indicating the most severe combined responses. PFIC and HIC showed intermediate separation with high SAA but relatively lower antioxidant activity. These patterns reflect the substantial effects of chemical and hormonal manipulations (51–53). CAT, GSH, and T-SOD projections anchor the protective antioxidant axis, with NC (untreated camels) and LBC (mechanically manipulated only) retaining higher antioxidant status and lower oxidative injury, confirming previous findings regarding the utility of these markers in assessing animal health and resilience. The reciprocal and opposing distribution of acute phase proteins and antioxidant enzymes substantiates their use as complementary indicators for evaluating camel welfare and the physiological consequences of illegal cosmetic manipulation interventions (13). Overall, PCA provides a robust synthesis of multidimensional data, enabling rapid stratification of welfare compromise and offering a quantitative framework for future biomarker-based animal welfare strategies in camels and other production species.

The heat map highlights a clear inverse relationship between antioxidant defenses and stress markers across camel cosmetic treatments. GSH and CAT cluster together, reflecting their coordinated antioxidant roles, while SAA separates from oxidative markers as an inflammation-specific protein. The gradient from NC to HIC and PFIC groups shows a dose–response effect of intervention severity, with LSC and LFIC in intermediate positions. This visualization approach provides a powerful tool for veterinary practitioners and welfare assessors to rapidly identify animals experiencing different degrees of physiological stress from cosmetic manipulations, supporting evidence-based regulatory enforcement and animal protection efforts.

This biomarker-based diagnostic framework establishes a practical pathway for implementation at major camel festivals and veterinary inspection protocols. The integration of serum biomarker analysis with existing detection methods (ultrasonography, thermography) creates a tiered diagnostic approach: initial screening via visual and ultrasonographic examination identifies suspected cosmetic alterations, followed by rapid point-of-care blood testing for SAA and oxidative stress markers to quantify physiological welfare compromise. Portable blood analyzer technologies, increasingly validated in field veterinary settings, enable on-site measurement of biomarkers, allowing real-time decision-making without requiring laboratory transport. Implementation would involve: (1) training veterinary personnel at festival venues in standardized blood sampling and point-of-care device operation; (2) establishing evidence-based threshold biomarker values triggering regulatory responses (e.g., SAA > 10 ng mL^−1^ or MDA > 3 nmol mL^−1^); (3) creating digital recording systems to document findings; and (4) integrating results into regulatory enforcement, enabling authorities to distinguish animals with objective evidence of severe physiological harm from those with minor cosmetic alterations. This approach harmonizes scientific rigor with practical feasibility, providing policymakers and veterinary inspectors with objective criteria to protect animal welfare while accommodating legitimate cultural practices through informed, evidence-based decision-making.

We acknowledge that assessing inflammation using only SAA does not capture the full spectrum of cellular and humoral immune responses. While this investigation provides the first comprehensive biomarker-based assessment of the effects of cosmetic tampering on dromedary camel welfare, several contextual factors merit consideration and offer clear opportunities for future research advancement. The study examined acute-phase inflammatory response using SAA, which effectively captures systemic inflammatory burden but represents a focused rather than exhaustive biomarker panel. This deliberate choice enabled a rigorous investigation of this critical marker while establishing a validated framework that future investigations can build upon. Complementary studies incorporating pro-inflammatory cytokines (IL-6, TNF-*α*), additional enzymatic antioxidants (GPx, GR), and direct oxidative damage markers (protein carbonyls, H₂O₂) would provide mechanistic depth and strengthen a multidimensional understanding of immune responses to cosmetic procedures. Similarly, the broad age distribution (1–20 years) across the festival population reflects real-world conditions; future studies employing tighter age stratification (e.g., 3–8 years representing young adults) or longitudinal age-cohort designs would enable more refined analysis and optimize statistical power through covariance adjustment. The cross-sectional design, while providing valuable baseline data on biomarker status during cosmetic procedures, establishes a crucial foundation for longitudinal investigations that track biomarker dynamics and recovery trajectories over time. Documentation of the interval between procedure execution and blood sampling in future work would clarify whether the observed elevations represent acute versus chronic responses, thereby further refining the temporal interpretation. Although the control group comprised festival animals, their inclusion provides an ecologically valid comparison that reflects natural conditions for handling animals. Future research could complement this approach by including non-festival control cohorts. These findings, while specific to dromedary camels at the mentioned Festival during a defined season, establish a quantitative, biomarker-based framework and proof-of-concept methodology applicable to other camelid species, geographic regions, and cultural contexts where cosmetic tampering practices occur.

## 5 Conclusion

This study demonstrates that all examined cosmetic procedures (LB, HI, LS, PFI, and LFI) significantly alter SAA and oxidative-stress biomarkers (MDA, CAT, SOD, and GSH). These changes are consistent with a systemic inflammatory and oxidative challenge in dromedary camels. The biomarker shifts correlate strongly and help quantify the degree of physiological compromise. Multivariate and clustering analyses clearly rank the severity of treatments, with hormonal and filler-based procedures causing the most severe redox disturbance, and mechanical manipulations alone also inducing considerable stress. Together, these findings provide the first objective, biomarker-based framework for assessing the welfare impact of camel aesthetic tampering, guiding evidence-based regulatory and veterinary decisions, and offering a scalable model for welfare assessment in other species exposed to practices that enhance performance or appearance. Future studies should track these biomarkers over time and add further redox and cytokine markers. Implementation of biomarker-based detection at major festival venues, combined with graduated regulatory standards informed by the severity of physiological harm, could substantially reduce welfare compromise while maintaining cultural practices. This research demonstrates that protection of animal welfare and respect for cultural traditions are not mutually exclusive; rather, objective evidence of harm enables communities to make informed decisions about sustainable, welfare-protective practices. The framework presented here applies to other culturally significant livestock practices globally, advancing international animal welfare standards based on measurable physiological science. These inferences are based on a focused biomarker panel (SAA and selected oxidative/antioxidant markers). Future studies incorporating cellular immune indices and additional redox biomarkers (e.g., GPx, GR, protein carbonyls, H₂O₂) are needed to characterize the immune-inflammatory response fully.

## Data Availability

The raw data supporting the conclusions of this article will be made available by the authors, without undue reservation.
