# Correction: Assessing animal welfare impacts of cosmetic manipulations in dromedary camels: insights from oxidative and inflammatory biomarkers

**DOI:** 10.3389/fvets.2026.1865095

**Published:** 2026-05-28

**Authors:** Mohamed Tharwat, Tariq I. Almundarij, Hassan Barakat

**Affiliations:** 1 Department of Clinical Sciences, College of Veterinary Medicine, Qassim University, Buraydah, Saudi Arabia; 2 Department of Medical Biosciences, College of Veterinary Medicine, Qassim University, Buraydah, Saudi Arabia; 3 Department of Food Science and Human Nutrition, College of Agriculture and Food, Qassim University, Buraydah, Saudi Arabia

**Keywords:** acute phase response, animal welfare, biomarkers, cosmetic procedures, dromedary camels, oxidative damage

There was a mistake in the caption of **Figure 3** as published.

In this caption, “Serum CAT was incorrectly written as “Serum ACT”. The corrected caption of Figure 3 appears below.

Figure 3. Serum CAT activity in camels subjected to different cosmetic procedures (mean ± SE). Serum CAT activity (U mL−1) was measured in camels following various treatments: NC, normal camels; LBC, lip binding camels; HIC, hormone injection camels; LSC, lip stretching camels; PFIC, perinasal filler injection camels; and LFIC, lip filler injection camels. Data are presented as mean ± standard error. ^a, b, c^Bars topped with different letters indicate statistically significant differences between treatments (*p*-value < 0.0001).

There was a mistake in Figures 4 and 5 as published. Figures 4 and 5 are a repetition of Figure 3. The corrected Figures 4 and 5 and their captions appears below.

**Figure 4:**
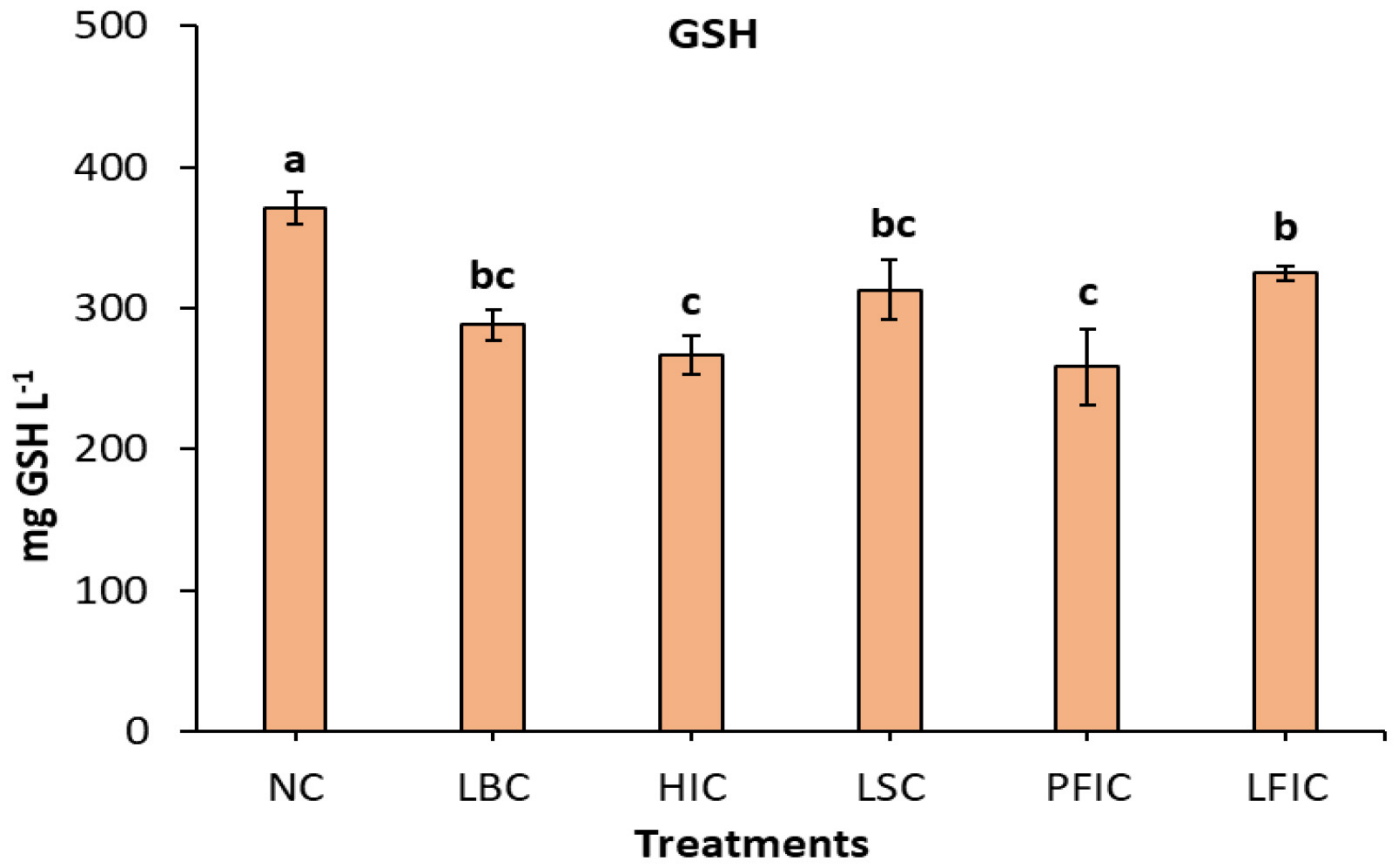
Serum GSH level in camels subjected to different cosmetic procedures (mean ± SE). Serum CAT activity (mg GSH mL−1) was measured in camels following various treatments: NC, normal camels; LBC, lip binding camels; HIC, hormone injection camels; LSC, lip stretching camels; PFIC, perinasal filler injection camels; and LFIC, lip filler injection camels. Data are presented as mean ± standard error. ^a, b, c^Bars topped with different letters indicate statistically significant differences between treatments (*p*-value < 0.0001).

**Figure 5:**
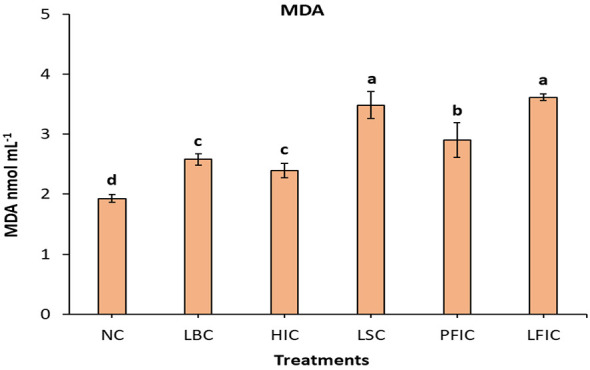
Serum MDA level in camels subjected to different cosmetic procedures (mean ± SE). Serum MDA (nmol mL−1) was measured in camels following various treatments: NC, normal camels; LBC, lip binding camels; HIC, hormone injection camels; LSC, lip stretching camels; PFIC, perinasal filler injection camels; and LFIC, lip filler injection camels. Data are presented as mean ± standard error. ^a, b, c^Bars topped with different letters indicate statistically significant differences between treatments (*p*-value < 0.0001).

The original version of this article has been updated.

