# Supplementary material for: Assessing animal welfare impacts of cosmetic manipulations in dromedary camels: insights from oxidative and inflammatory biomarkers
Source: Front Vet Sci. 2026 Mar 16;13:1720235. doi: 10.3389/fvets.2026.1720235 (PMC13033491; doi:10.3389/fvets.2026.1720235)
Supplement: Supplementary file 1 [file Data_Sheet_1.PDF]

Table S1. PCA Loadings

Biomarker	PC1	PC2	PC3	PC4	PC5
**SAA**	+0.935	0.001	0.355	-0.007	0.000
**CAT**	-0.851	0.514	0.107	-0.016	0.007
**T-SOD**	-0.892	-0.417	0.129	0.115	-0.001
**GSH**	-0.882	0.457	0.103	-0.046	-0.007
**MDA**	0.579	0.807	-0.060	0.096	-0.001

Figure S1. Scattering plots of the means of examined biomarkers (SAA, CAT, T-SOD, GSH, MDA)

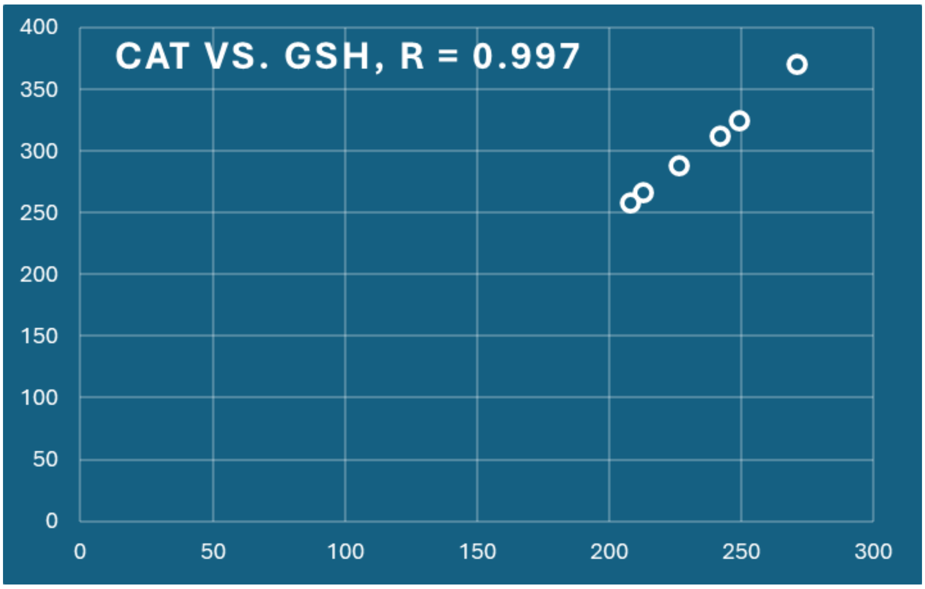

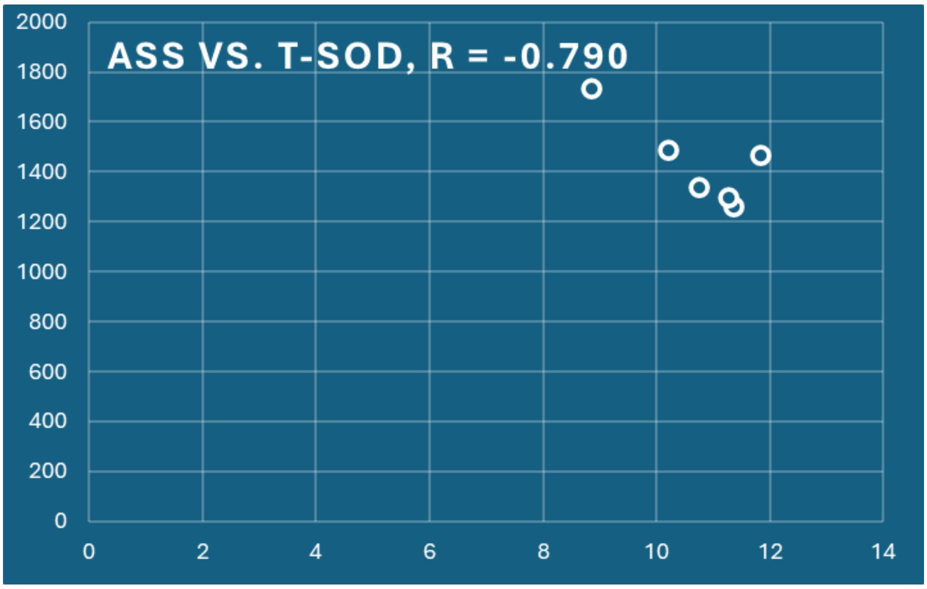

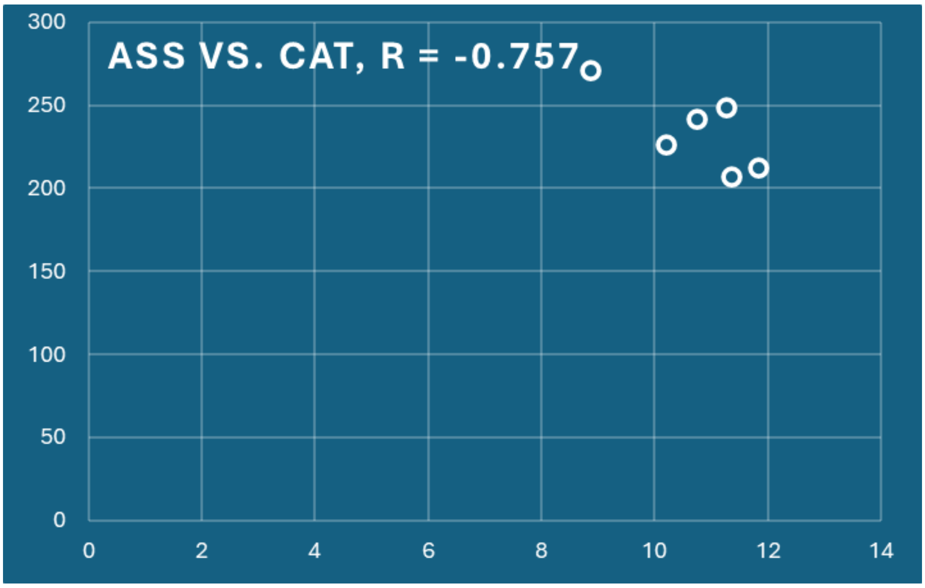
ASS VS. CAT, R = -0.757
300
250
200
150
100
50
0
0
2
4
6
8
10
12
14

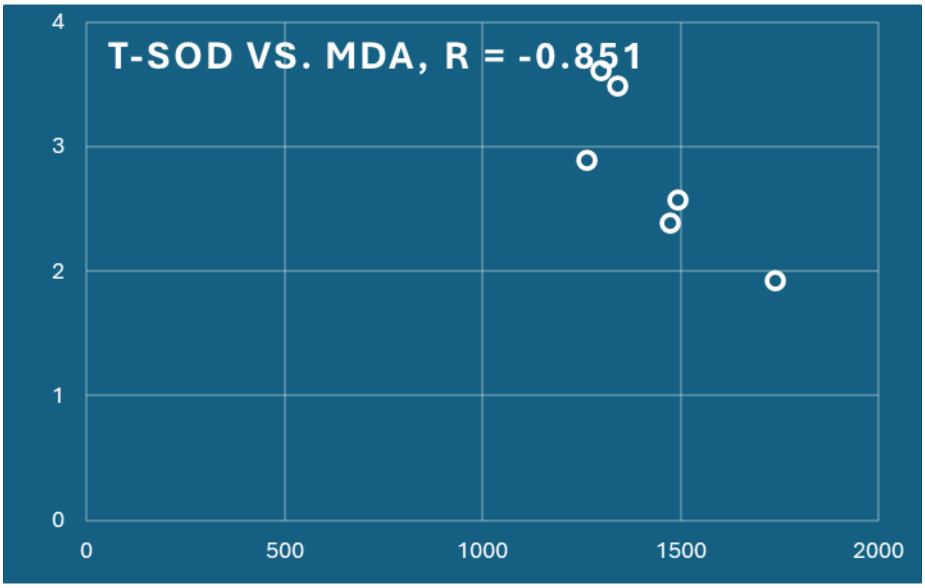
T-SOD VS. MDA, R = -0.851
4
3
2
1
0
0
500
1000
1500
2000

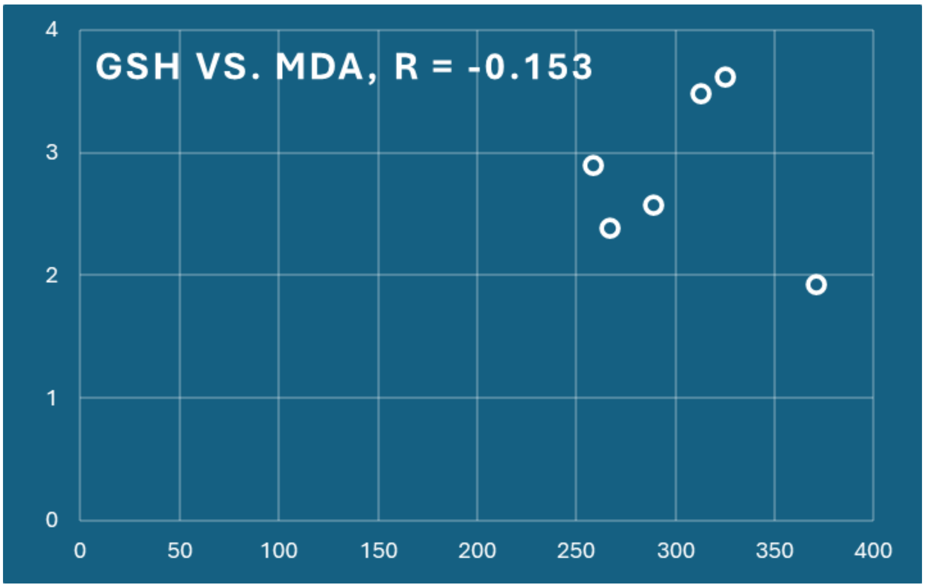
GSH VS. MDA, R = -0.153
4
3
2
1
0
0
50
100
150
200
250
300
350
400
